# Numerical Simulation and Experimental Study of Carbon Fiber-Reinforced Polymer Single-Bar Extrusion Anchorage Structure

**DOI:** 10.3390/ma17163915

**Published:** 2024-08-07

**Authors:** Wanxu Zhu, Chengyang Xiong, Boxuan Cheng, Quanxi Shen, Hongbin Cheng, Shangqi Guo

**Affiliations:** 1 School of Civil Engineering, Guilin University of Technology, Guilin 541004, China; zhuwanxu@vip.163.com (W.Z.); xiongcy0922@163.com (C.X.); chenbx1996@163.com (B.C.); shenquanxi12@163.com (Q.S.); tcchb0325@163.com (H.C.); 2 China Nonferrous Metals (Guilin) Geology and Mining Co., Ltd., Guilin 541004, China; 3 College of Earth Sciences, Guilin University of Technology, Guilin 541004, China; 4 Guangxi Hanximing Technology Co., Ltd., Guilin 541004, China

**Keywords:** carbon fiber-reinforced polymer tendons, extrusion-type anchor, anchorage structure, static load test, finite element analysis

## Abstract

The reliable anchorage of carbon fiber-reinforced polymer (CFRP) tendons is a critical issue influencing the stable bearing capacity of bridge cables. This study introduces a novel CFRP single-strand extrusion anchoring structure, where the strand is compressed at its end. By integrating this with internal cone filler wrapping, we create a CFRP multi-strand cable composite anchoring system. This innovative design not only minimizes the overall dimensions of the anchoring system but also significantly improves its anchoring efficiency coefficient. An axisymmetric model was developed using ANSYS finite element software. The radial stress distribution and anchorage efficiency coefficient in the anchorage zone of Φ7 CFRP bar and Φ13.6 extrusion die were analyzed with varying parameters, such as chamfering, outer diameter, and length of the extrusion sleeve, and were validated through static load anchorage tests. The results indicate that the highest anchoring efficiency is achieved when four extrusion sleeves with a chamfer angle of 5°, an outer diameter of Φ14.4, and a length of 15 mm are connected in series, reaching a coefficient of 61.04%. Furthermore, this study proposes an anchorage structure where multiple extrusion sleeves are connected in series and sequentially compressed to overcome the limitations of increasing anchorage length for enhancing the anchorage coefficient. The test results demonstrate that with equal total anchorage length, connecting four 15 mm extrusion sleeves in series enhances the anchorage efficiency coefficient by 24.98% compared to a single 60 mm extrusion sleeve structure.

## 1. Introduction

Currently, high-strength steel strands or wires predominantly serve as load-bearing materials for large cable structures. However, corrosion issues with steel can precipitate the premature failure of the entire structure [1], significantly reducing the service life of engineering structures [2] and escalating maintenance costs [3]. Carbon fiber-reinforced polymer (CFRP) tendons, characterized by their light weight, high strength, fatigue resistance, and corrosion resistance [4,5], are anticipated to address the issues of self-weight and poor durability associated with steel cables [6]. Additionally, CFRP tendons are expected to enhance the spanning capacity of bridges [7], representing a future development direction for cable-stayed bridges. Nonetheless, ensuring the reliable anchorage of CFRP tendons remains an urgent challenge that needs to be addressed in engineering applications.

**Figure 1:**
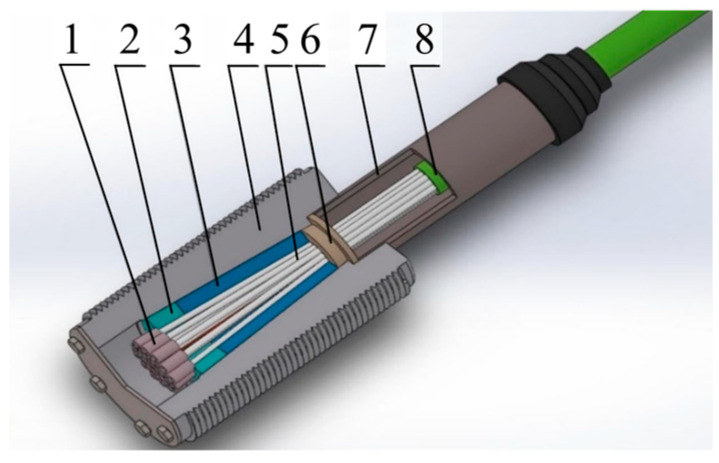
CFRP multi-bundle cable composite anchorage structure. 1 Extrusion sleeve; 2 splitting plate; 3 load transfer medium; 4 anchor cup; 5 CFRP tendons; 6 constraint circle; 7 extended anchor tube; 8 cable body.

At present, the commonly used CFRP tendon anchorage at home and abroad can be summarized into the following four forms: clip type, adhesive type, composite type, and extrusion type [8,9]. Wedge-type anchorage has been widely studied because of its advantages of simple installation and reusability [10]. However, wedge-type anchorage is mainly suitable for anchoring single reinforcement [11], and the slip of the anchorage is large [12]. At the same time, it is easy for the wedge to cause damage to the reinforcement [13]. Bonded anchorage is suitable for the anchorage of multiple CFRP tendons [14]. It does less damage to the reinforcement [15,16] and has been applied to practical engineering [17,18]. However, there are problems, such as the loss of bonding force caused by the aging of the bonding medium [19] in the harsh working environment, and the durability, which is difficult to guarantee. At present, most composite anchorages are composited with bonded anchorages and clip-type anchorages [20,21], which still need to rely on the bonding anchorage force [22], and the durability is questionable. The extrusion anchorage is divided into two forms: internal expansion extrusion and mechanical extrusion. The internal expansion extrusion method has been successfully applied to many bridges made by Japan CFCC and with Mitsubishi Chemical’s Leadine cable [23], but the expansion material will be aged and damaged in long-term loading and complex environments, and its fatigue resistance and durability are difficult to guarantee. Mechanical extrusion anchorage uses a metal sleeve to press on a single CFRP bar through an extruder. By adjusting the outer diameter and length of the metal sleeve, a higher anchorage efficiency coefficient can be achieved. Although it is only suitable for single-bar anchorage, Zhu [24] proposed a non-bonded composite anchorage structure combining LTM and single-bar extrusion, as shown in Figure 1. By adding an extrusion sleeve at the end of the reinforcement, it can not only share part of the axial load and serve as a ‘insurance line’ to prevent the slippage of the reinforcement, but also improve the anchorage efficiency coefficient while reducing the size of the entire anchorage structure. Due to the sudden change in stiffness between the extruded anchorage structure and the reinforcement, it is easy to cause damage to the CFRP tendons with weak lateral strength, and a slight assembly deviation can easily cause fracture. In recent years, research on the extruded anchorage structure of CFRP tendons has been scarce. Liuzhou OVM Co., Ltd. developed a CFRP tendon extrusion-bonding composite anchor [25], achieving high anchoring force without damaging the tendon. The extrusion sleeve is designed to increase the contact area between the tendon and the bonding medium and does not directly bear the load. Therefore, the impact of the extrusion sleeve on the tendon’s load-bearing capacity was not studied in depth. Wang [26] and Sun [27] proposed a method to anchor Φ12 CFRP tendons using aluminum alloy short tubes through hydraulic clamping and extrusion and investigated the effects of extrusion sleeve length and depth on anchoring efficiency. However, the method’s large transverse dimensions make it unsuitable for multi-strand cable anchors and result in high slip and low anchoring efficiency. Due to the narrow internal space of the anchor cup, the outer diameter of the extrusion sleeve is limited. At the same time, when the extrusion force is too large, it is easy to cause the reinforcement to bend and damage the reinforcement. Therefore, it is imperative to develop a CFRP tendon extrusion sleeve with the length and outer diameter as small as possible, but still meeting the anchorage requirements.

This study presents a finite element model of the extrusion anchorage process for Φ7 CFRP bars utilizing ANSYS software ANSYS19.2. By varying parameters such as chamfer, outer diameter, and sleeve length, an optimal distribution of extrusion stress is achieved. The design, refined through static load test results, addresses a theoretical gap in understanding the mechanical extrusion anchorage of single CFRP bars. Furthermore, this study introduces the novel use of multiple extrusion sleeves in series to enhance anchorage. This approach mitigates the limitations of single-sleeve anchoring force and prevents bending issues during installation caused by excessive sleeve length. It is demonstrated for the first time that over 61.04% of the anchoring force can be distributed without damaging the bar material, offering a new design foundation for practical applications of CFRP bar extrusion anchorage systems.

## 2. Test Survey

The composite extrusion anchor at the terminal of the bonded anchorage structure of CFRP tendons, serving as a bearing unit, enhances the anti-slip performance and significantly improves the anchorage efficiency coefficient through the plastic deformation of the anchorage bar. The extrusion sleeve has a small volume, and its load-sharing capability can shorten the anchorage section length, thereby reducing the overall size of the anchorage. The performance requirement for the extrusion sleeve stipulates that load sharing should not exceed 40% of the reinforcement’s ultimate force. Considering a safety factor of 1.2, the target value for the anchorage performance of a single reinforcement extrusion anchor is 48%.

### 2.1. Specimen Design

**Figure 2:**
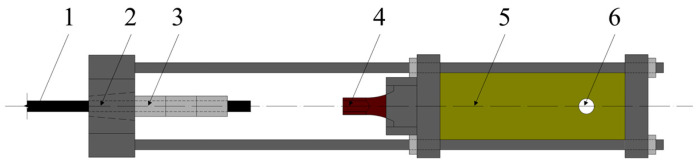
Installation structure diagram of CFRP tendon tandem extrusion anchorage. 1—CFRP tendon; 2—extruding anchor; 3—extrusion sleeve; 4—top pressure head; 5—jack; 6—tubing and oil pump.

**Figure 3:**
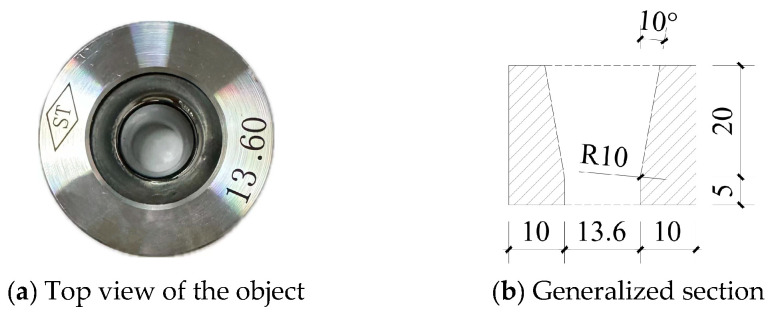
Physical drawing and schematic drawing of extrusion die.

**Figure 4:**
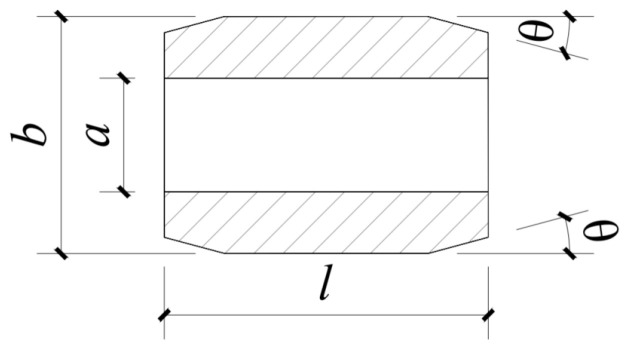
Squeeze sleeve structure diagram. *a*—The inner diameter of the extrusion sleeve; *b*—The outer diameter of the extrusion sleeve; *l*—The length of the extrusion sleeve; *θ*—The chamfer angle of the extrusion sleeve.

**Figure 5:**
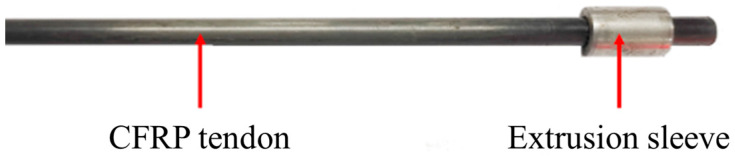
Physical drawing of CFRP tendons and extrusion anchor.

The extrusion anchorage specimen designed in this study consists of three components: CFRP tendons, extrusion sleeves, and an extrusion die (extrusion machine). The CFRP tendons used are smooth bars with a strength grade of 2400 MPa and a diameter of Φ7. The composite tendons are threaded through the central installation hole of the pressure plate. Multiple extrusion sleeves, lubricated and linked in series, are then threaded through the composite tendons, which are inserted into the pressure head. The jack is operated to perform the extrusion anchorage. Figure 2 shows the installation structure of the CFRP tendon series extrusion anchor, with Figure 3 providing a top view and sectional schematic of the extrusion die, and Figure 4 illustrating the structure of the extrusion sleeve. During the extrusion process, the extrusion sleeve undergoes plastic deformation, generating radial stress to anchor the CFRP tendons. The actual anchored specimen is shown in Figure 5.

**Figure 6:**
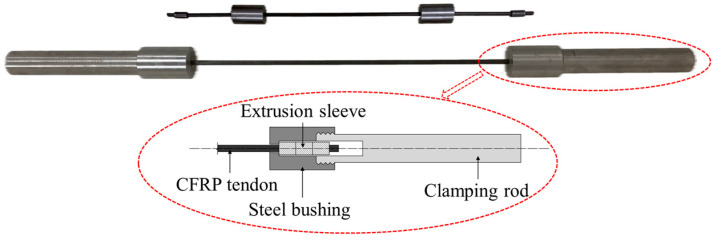
Auxiliary members for static load test of CFRP bar extrusion anchorage structure.

The CFRP specimens were manufactured in compliance with GB/T 30022-2013 [28] standards. To ensure that the test results were not influenced by the grips of the testing machine, the CFRP bar extrusion anchorage structure was loaded using a specialized testing device. A photograph of the manufactured specimens is presented in Figure 6.

**Figure 7:**
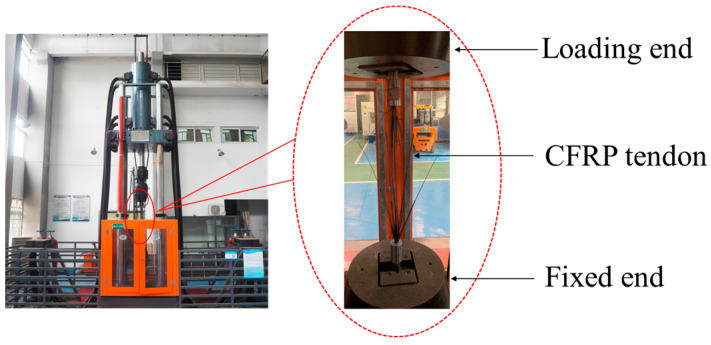
Electro-hydraulic servo universal testing machine.

The testing instrument utilized is an electro-hydraulic servo universal testing machine to test the CFRP tendon specimens. Figure 7 shows the actual testing machine and the specimens mounted on the machine. The test procedure adhered to GB/T 1446-2005 [29] standards, starting with an initial preload of up to 5 kN to eliminate initial displacement and bending moments caused during specimen installation, thereby reducing the impact of installation deviations on the test results. Subsequently, the specimens were subjected to displacement-controlled loading at a rate of 2 mm/min until failure.

### 2.2. Material Mechanical

CFRP reinforcement utilizes Φ7 round bars, exhibiting mechanical anisotropy. The extrusion die material selected is Cr12MoV (ASTM D2) tool steel. The extrusion sleeve material chosen is 45# steel (ASTM 1045) with a yield strength of 355 MPa. See Table 1 [30] for the properties of each component material.

## 3. Pressure Adjustment Mechanism for CFRP Tendon Anchorage Structures

**Figure 8:**
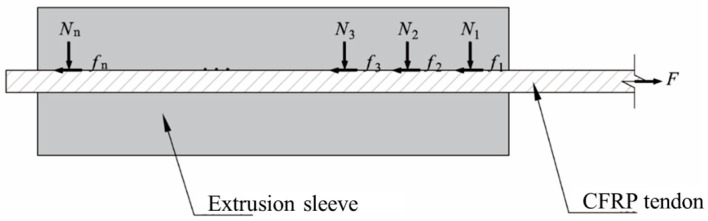
Schematic diagram of the force analysis of the anchorage structure.

The design of the anchoring structure must satisfy the equation $\sigma_{1}-K\sigma_{2}\leq X_{t}$ [24] to ensure that CFRP tendons do not fail due to rupture within the anchorage zone. Using the infinitesimal element method, the extruded anchorage structure is divided into n segments, numbered from the loaded end to the free end. Each segment of the CFRP tendon is subjected to radial compressive force from the extrusion sleeve and axial friction, as shown in Figure 8.

Analyzing each infinitesimal segment separately, it is challenging to control the compressive force uniformly during the extrusion process. Therefore, to ensure structural safety, the relationship between tensile and compressive stresses at any infinitesimal segment *i* of the extruded anchorage structure must comply with Equation (1).

(1) $$k_{1}\bar{\sigma_{1i}}-Kk_{2}\bar{\sigma_{2i}}\leq X_{t}$$

where $\bar{\sigma_{1i}}$—the average tensile stress of microelement *i* in the tendon anchorage zone;

$\bar{\sigma_{2i}}$—the average compressive stress of microelement *i* in the tendon anchorage zone;

$k_{1}$—the tensile stress concentration factor of microelement *i* in the tendon anchorage zone;

$k_{2}$—the compressive stress concentration factor of microelement *i* in the tendon anchorage zone;

*K*—the safety factor of the tendon; and

$X_{t}$—the axial tensile strength of the tendon.

To fully utilize the tensile strength of CFRP tendons, each infinitesimal segment of the tendon should be in an equal failure state. That is, each segment *i* must simultaneously satisfy Equation (2). Thus, for any segment *i* and *j*, the following applies:

(2) $$k_{1}\bar{\sigma_{1i}}-Kk_{2}\bar{\sigma_{2i}}=k_{1}\bar{\sigma_{1j}}-Kk_{2}\bar{\sigma_{2j}},i,j=1,2,3,\ldots ,n$$

Based on the load-bearing characteristics of the anchorage structure, friction between the extrusion sleeve and the tendon causes the tensile stress within each infinitesimal segment to gradually decrease along the axial direction from the loaded end to the free end. Due to the high modulus of elasticity of CFRP tendons, the change in cross-sectional area caused by anchor compression can be neglected. Consequently, it can be concluded that

(3) $$k_{1}\left(F-\sum_{n=1}^{i-1}F_{n}\right)-\frac{Kk_{2}nd}{4l}N_{i}=k_{1}\left(F-\sum_{n=1}^{j-1}F_{n}\right)-\frac{Kk_{2}nd}{4l}N_{j}$$

where *l*—the length of the anchorage zone; and

*d*—the diameter of the tendon.

The closer a cross-section of the anchorage structure is to the loaded end, the greater the axial tensile force it experiences. Thus, it can be concluded that

(4) $$N_{n}>N_{\left(n-1\right)}>\ldots >N_{2}>N_{1}$$

Thus, for the extruded anchorage structure to achieve a high anchorage efficiency coefficient, the compressive force on the tendon should gradually increase from the loaded end to the free end. However, in practical applications, due to construction deviations or limitations in the structure’s dimensions, it is challenging to ensure uniform strength across all segments of the anchorage structure. Whether in static load or fatigue tests, failure often occurs at the loaded end of the anchorage structure. For economic considerations, attention should be focused on the weak points of the anchorage structure, such as reducing the stress level at the loaded end.

## 4. Finite Element Module Method

This paper extensively investigates the stress characteristics of CFRP bar anchorages using numerical simulation, focusing particularly on analyzing factors influencing the radial compressive stress distribution in the anchorage zone. The aim is to derive more rational design solutions. Based on the structural characteristics of the extrusion anchor, a finite element model of the extrusion sleeve pressing process has been developed. This model utilizes structural axisymmetry to simplify the three-dimensional problem into a two-dimensional axisymmetric model.

### 4.1. Element Selection

This study conducts finite element analysis on the specimens using ANSYS software. The tendons, extrusion die, and extrusion sleeves are modeled using Solid 183 elements, with sweep meshing applied. The contact behavior between the tendons and the extrusion sleeve, as well as between the extrusion die and the extrusion sleeve, was modeled using the Augmented Lagrangian Contact Algorithm, as detailed in Equation (5). A standard contact behavior model was established. The contact stiffness is set to soft–soft contact, and both contact interfaces are simulated using surface-to-surface contact, with the contact surface modeled using CONTA 172 elements and the target surface using TARGE 169 elements.

(5) $$F_{Normal}=k_{Normal}x_{Penetration}+\lambda$$

where $F_{Normal}$—contact force;

$k_{Normal}$—contact stiffness;

$x_{Penetration}$—penetration; and

λ—Lagrangian multiplier.

The friction coefficient between the tendons and the extrusion sleeves is set to 0.23 [31]. Due to the application of lubricants like anti-seize agent in the extrusion die during extrusion, the friction coefficient between the die and sleeves is set very low, at 0.04.

### 4.2. Finite Element Model

**Figure 9:**
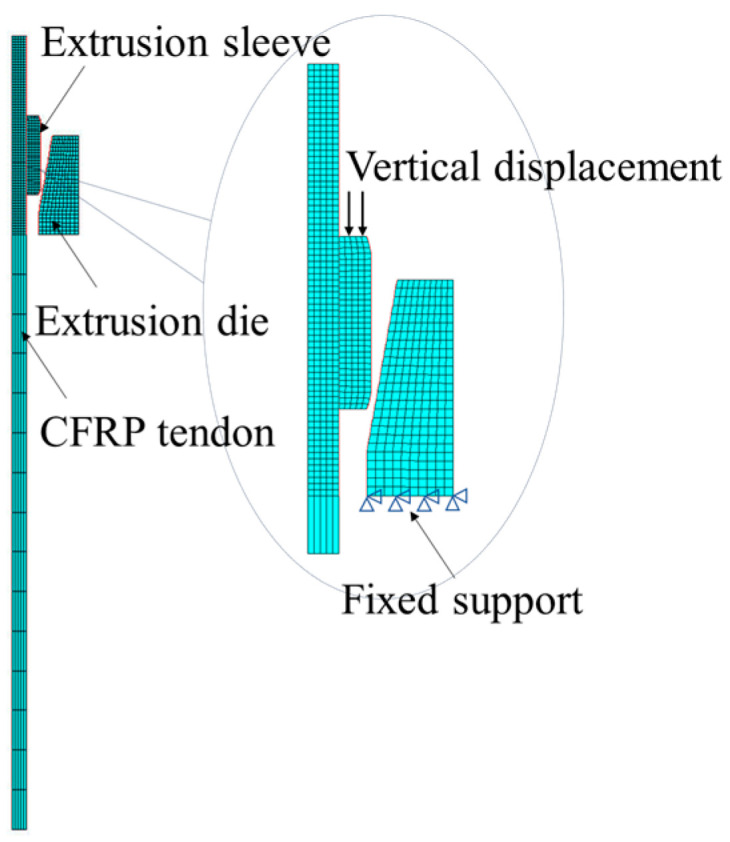
Finite element model of specimen.

The model employs a distinct methodological approach by developing an axisymmetric finite element model of the extrusion anchor, utilizing the precise dimensions of the test specimens at a 1:1 scale, as illustrated in Figure 9. The CFRP bar model is characterized by a length of 200 mm and a radius of 3.5 mm; the extrusion sleeve model features a chamfer angle of 20°, an outer diameter of 14.6 mm, and a length of 20 mm; and the extrusion die model is defined by a lower edge length of 10 mm, a height of 25 mm, and a tilt angle of 10°, as detailed in Figure 3b.

The size of the mesh elements during the division process markedly influences the convergence and accuracy of the finite element model’s results. Trial calculations with varying mesh sizes revealed that when the mesh elements are nearly square with an edge length of less than 1 mm, the relative error in the simulation remains below 5%, although the computation time increases substantially. Conversely, when the mesh edge length exceeds 1 mm, the computation time decreases, but there is a considerable reduction in accuracy. Consequently, in this model, the end of the CFRP bar contains 70 longitudinal and 5 transverse mesh elements in the initial 50 mm segment, and 15 longitudinal and 5 transverse mesh elements in the subsequent 150 mm segment, resulting in a total of 425 mesh elements. The extrusion die features 25 longitudinal and 10 transverse mesh elements, resulting in a total of 250 mesh elements.

The loading and boundary conditions of the model are shown in Figure 9. A vertical displacement is applied to the top of the extrusion sleeve, and the extrusion die is fixed with boundary constraints.

### 4.3. The Constitutive Model of Extrusion Sleeve and Extrusion Die Material

**Figure 10:**
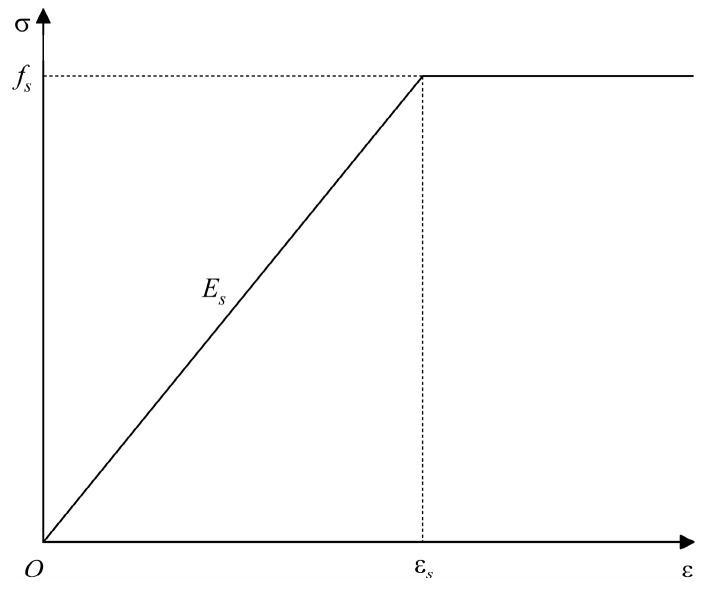
Constitutive material of extrusion sleeve.

During the extrusion anchoring process of CFRP tendons, the extrusion die remains in an elastic state and does not undergo plastic deformation, while the extrusion sleeve experiences significant plastic deformation. To simplify the analysis, the extrusion die is considered an ideal linear elastic material, whereas the plastic deformation of the extrusion sleeve is taken into account, viewing it as an ideal elastoplastic material. The material constitutive relationship is shown in Figure 10.

## 5. Refined Numerical Simulation Analysis

By analyzing the deformation of the extrusion anchor and the radial stress distribution of the tendon material after installing the extrusion sleeve, the influence of parameters such as the chamfer angle, the outer diameter, and the length on the radial stress distribution in the anchoring area of the tendon is determined, providing more reliable theoretical guidance for CFRP tendon extrusion anchoring structures.

### 5.1. Chamfer Angle Analysis of Extrusion Sleeve

Based on experience, a preliminary selection of a 20 mm long extrusion sleeve with an outer diameter of Φ14.6 is made, corresponding to an extrusion die with an inner diameter of Φ13.6. The CFRP tendon uses a Φ7 smooth round bar, and finite element models are established by adjusting the chamfer angles at both ends of the extrusion sleeve.

**Figure 11:**
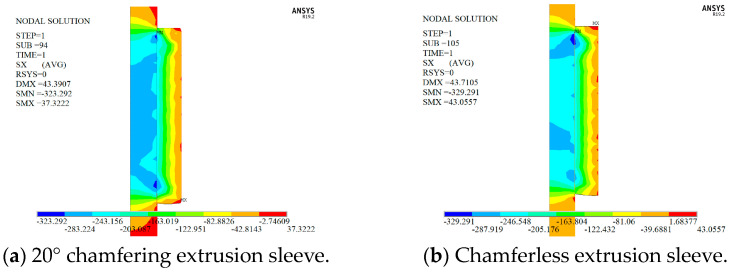
Radial stress cloud diagram of extrusion anchor.

To determine whether the presence of chamfers at both ends of the extrusion sleeve can improve the radial stress distribution of the tendon material, models were created for the extrusion process with and without chamfers. The numerical simulation results are shown in Figure 11. Figure 11a corresponds to the extrusion sleeve with a 20° chamfer at both ends, while Figure 11b shows the extrusion sleeve without chamfers, with the free end at the top and the loaded end at the bottom.

**Figure 12:**
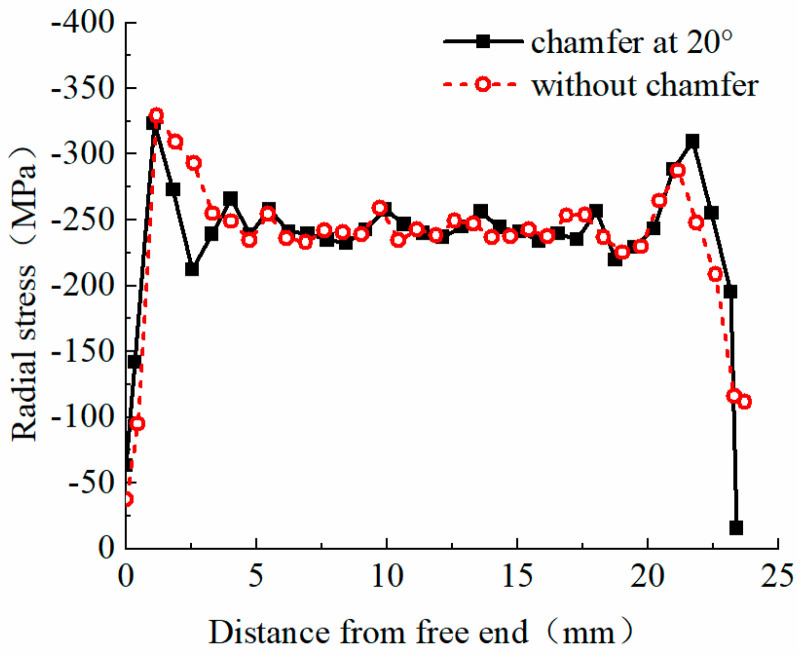
Radial stress distribution curve of anchorage zone of reinforcement.

As shown in Figure 12, significant radial stress concentrations occur at both ends of the extrusion sleeve anchoring zone, with lower stress in the center and peak stress near the free end. Lateral compressive stress negatively impacts the load-bearing capacity of the reinforcing bars under axial tension. Therefore, analyzing the positions at both ends of the anchoring zone is essential. Frictional forces within the extrusion sleeve reduce the axial stress at the free end by approximately 30% compared to the loaded end, while the lateral stress at the free end increases by up to 15%. For structural safety, prioritizing the loaded end in anchoring structure design is necessary.

Figure 12 shows that the peak radial stress at the loaded end of the extrusion anchor with a 20° chamfer increases by 22.24 MPa compared to the non-chamfered anchor, with values of −309.76 MPa and −287.52 MPa, respectively. This appears counterintuitive. Thus, a thorough investigation into the chamfer’s effect on radial stress is needed to evaluate the chamfer setting’s reasonableness and to determine the optimal angle.

**Figure 13:**
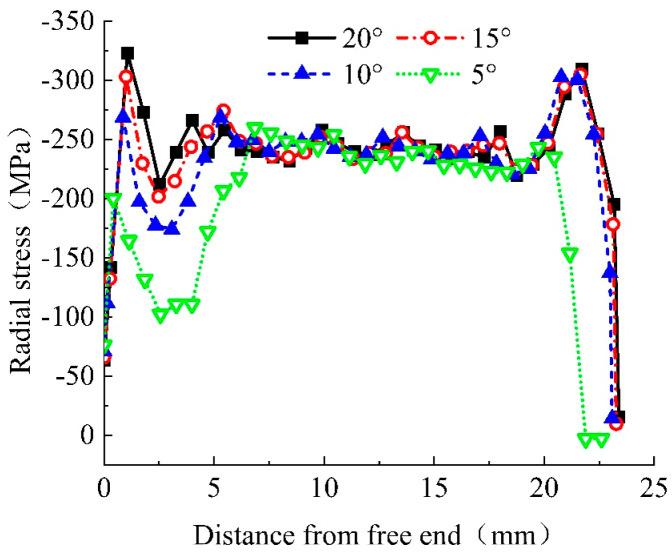
Radial stress distribution curve of anchorage zone of each chamfered extrusion anchor bar.

Finite element models with chamfer angles of 5°, 10°, 15°, and 20° were established for further analysis. Figure 13 shows the radial stress distribution curves of the reinforcing bars within the extrusion anchor range. Varying the chamfer angle significantly affects the radial stress values in the anchoring zone.

By analyzing the radial stress distribution curves of the anchoring area of extrusion anchors with different chamfer angles, it is found that after extrusion, the maximum radial stress remains concentrated near the free end. Reducing the chamfer angle moves the maximum stress point inward, closer to the center of the anchoring area. Additionally, decreasing the chamfer angle shortens the anchoring area, which aligns with intuitive understanding. Smaller chamfer angles also result in lower peak radial stress at the loaded end. For chamfer angles of 10°, 15°, and 20°, the peak radial stresses near the loaded end are −302.97 MPa, −304.87 MPa, and −309.76 MPa, respectively, differing by about 2%. However, the 5° chamfer angle results in a peak radial stress of −243.23 MPa, which is 21.5% lower than the 20° chamfer angle, indicating a significant difference from the other three angles. Chamfering at the loaded end is crucial, as it effectively reduces radial stress values at this section of the reinforcing bars, preventing the “notch effect”.

**Figure 14:**
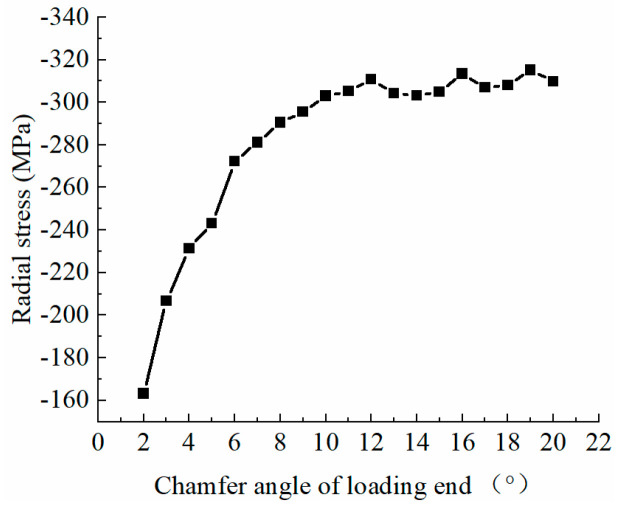
The relationship between the peak radial stress at the loading end of the stiffener and the chamfering angle of the loading end of the extrusion sleeve.

To further understand the chamfer angle’s effect on peak radial stress at the loaded end, and to determine the optimal chamfer angle for the extrusion sleeve, models with chamfer angles from 2° to 20° were established. Figure 14 illustrates the relationship between peak radial stress at the loaded end of the reinforcing bars and the extrusion sleeve’s chamfer angle. When the chamfer angle exceeds 10°, the difference in peak radial stress at the loaded end is minimal. Conversely, when the chamfer angle is less than 10°, the peak radial stress at the loaded end decreases rapidly with decreasing chamfer angle.

**Figure 15:**
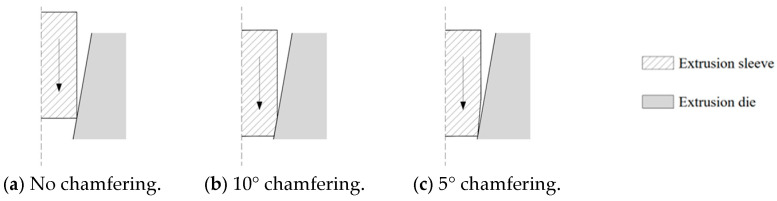
Contact conditions between extrusion sleeve and extrusion die.

Analyzing the contact between the extrusion sleeve and the extrusion die reveals that when the chamfer angle of the loaded end of the extrusion sleeve exceeds the taper of the die’s inner hole, as shown in Figure 15a, the sleeve begins to contact the die as it is pushed down before fully entering the hole. The presence of friction prevents downward plastic deformation, leading to high radial stress at the loaded end. When the chamfer angle of the sleeve is approximately equal to the die’s taper angle, as shown in Figure 15b, plastic deformation is restricted during downward movement, also resulting in high residual stress. Conversely, when the chamfer angle is less than the die’s taper angle, as illustrated in Figure 15c, a gap exists at the upper contact surface, allowing upward plastic deformation due to friction, which reduces peak radial stress. Therefore, to effectively prevent the “notch effect” at the loaded end of the bar, the chamfer angle of the extrusion sleeve should be smaller than the die’s taper angle, with smaller angles being preferable.

However, in practical engineering applications, the extrusion sleeve’s length and outer diameter are often constrained by design and construction considerations, limiting the reduction in the chamfer angles. A chamfer angle of 5° is generally recommended.

### 5.2. Analysis of Outer Diameter of Extrusion Sleeve

The outer diameter of the extrusion sleeve is crucial for the anchoring efficiency coefficient of the extrusion anchor. The anchoring force depends on the plastic deformation of the extrusion sleeve; hence, a larger outer diameter provides a greater extrusion margin and results in more significant plastic deformation during extrusion.

**Figure 16:**
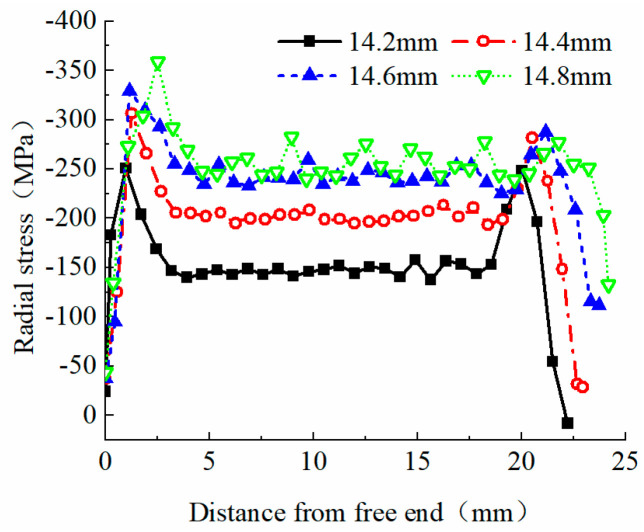
The radial stress distribution curve of each outer diameter extrusion anchor bar material.

For an extrusion sleeve length of 20 mm with no chamfers at both ends, finite element models were established for outer diameters of Φ14.2, 14.4, 14.6, and 14.8. The radial stress distribution maps were obtained, and the radial stress distribution curves of the reinforcing bars are plotted as shown in Figure 16. As the outer diameter of the extrusion sleeve increases, the anchoring zone of the reinforcing bars extends, and the radial stress increases correspondingly, although the distribution patterns remain similar.

Theoretical analysis indicates that minimizing the radial stress at the loaded end is crucial for the safety of the anchoring structure. Therefore, a Φ14.2 extrusion sleeve is preferable, though its lower average radial stress reduces the anchoring efficiency coefficient. Conversely, the average radial stress of Φ14.4 and Φ14.6 sleeves increases by 29.6% and 55.5%, respectively, compared to Φ14.2, with corresponding increases of 9.0% and 12.9% in maximum radial stress at the loaded end. The Φ14.8 sleeve further increases the average radial stress by 6.6% and the maximum radial stress by 5.1%, compared to Φ14.6. Thus, an outer diameter of Φ14.4 to Φ14.6 is recommended for optimal structural safety and anchoring efficiency.

### 5.3. Extrusion Sleeve Length Analysis

The length of the extrusion sleeve determines the post-extrusion anchorage zone length. A longer anchorage zone offers greater anchorage force to the reinforcement material, enhancing the extrusion anchor’s efficiency coefficient.

**Figure 17:**
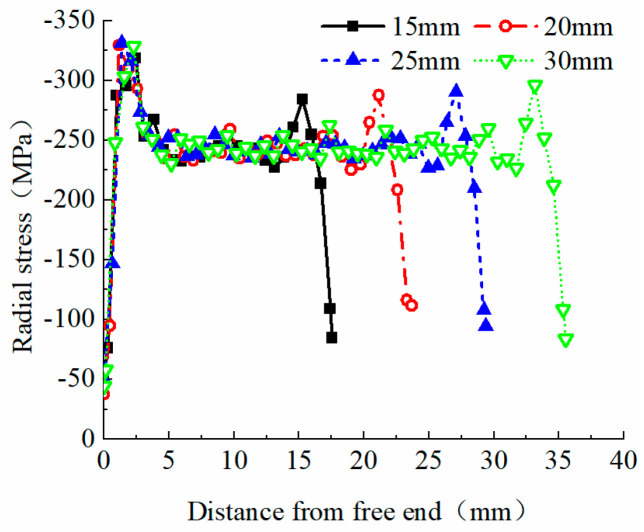
Radial stress distribution curve of each length of extruded anchor bar material.

Finite element models were established for extrusion sleeves with an outer diameter of Φ14.6 and no chamfers at both ends, with lengths of 15 mm, 20 mm, 25 mm, and 30 mm. The radial stress distribution diagrams and curves for the reinforcement material are shown in Figure 17. As the length of the extrusion sleeve increases, the radial stress in the middle of the anchorage zone remains stable around −250 MPa. The maximum radial stress near the load-bearing end stays relatively stable around −290 MPa, with fluctuations of up to 5%.

Theoretically, increasing the sleeve length could enhance anchorage effectiveness. However, in practical applications, factors such as the precision of the extrusion machine and the surface smoothness of both the extrusion sleeve and die can significantly affect the stability of the extrusion anchor. Particularly when the sleeve is long, it can lead to issues such as tendon bending or shearing. Therefore, it is crucial to minimize these adverse effects during construction to prevent tendon damage.

**Figure 18:**
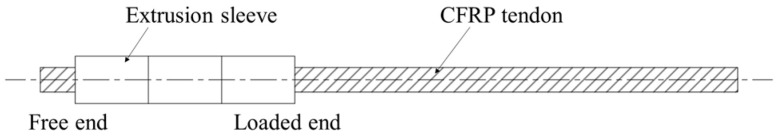
Schematic diagram of CFRP tendon series extrusion anchorage structure.

This paper proposes a series extrusion anchor scheme to solve this issue. By limiting the length of the individual extrusion sleeves and connecting multiple sleeves in series, the total anchorage zone length can be achieved without excessive sleeve length. As shown in Figure 18, each sleeve is tightly pressed together during extrusion, collaboratively anchoring the tendon. This approach ensures the required total anchorage length is met while effectively preventing tendon damage due to too-long extrusion sleeves.

## 6. Static Load Tensile Test

Based on the previous finite element analysis results, this study aims to investigate the influence of various parameters of the extrusion sleeve on the anchorage performance of anchors and to validate the anchorage performance of the newly designed CFRP extrusion-type anchor. Corresponding specifications of the extrusion anchor were selected for static tensile testing of CFRP single tendon extrusion anchors.

According to relevant standards [32], the static anchorage performance of CFRP tendons should meet the following criteria:

(1) The failure mode in the static load test of the CFRP tendon anchorage structure should be the failure of the tendon, not the failure of the anchor.

(2) The anchorage efficiency coefficient determines the static anchorage performance of the anchorage structure. It is calculated as follows:

(6) $$\eta_{a}=\frac{F_{\mathrm{Tu}}}{F_{\mathrm{ptk}}}\geq 90\%$$

where $\eta_{a}$—anchorage efficiency coefficient (%);

$F_{\mathrm{Tu}}$—the measured ultimate tensile force of CFRP tendon anchorage structure (kN); and

$F_{\mathrm{ptk}}$—the nominal ultimate tensile strength of CFRP tendons (kN).

By conducting static tensile tests on the CFRP tendon anchorage structure, the anchorage efficiency coefficient of the newly designed extrusion-type anchor can be determined, quantifying its anchorage performance. This, combined with the previous finite element analysis results, allows for the comparison and validation of the effects of various parameters of the extrusion sleeve on the anchorage performance.

### 6.1. Static Load Tensile Test of Single Extrusion Sleeve Anchorage CFRP Tendon

Based on finite element analysis conclusions, 12 specimens with different extrusion sleeve parameters were designed for static tensile tests, as shown in Table 2. Three identical specimens were made for each set of parameters.

**Figure 19:**
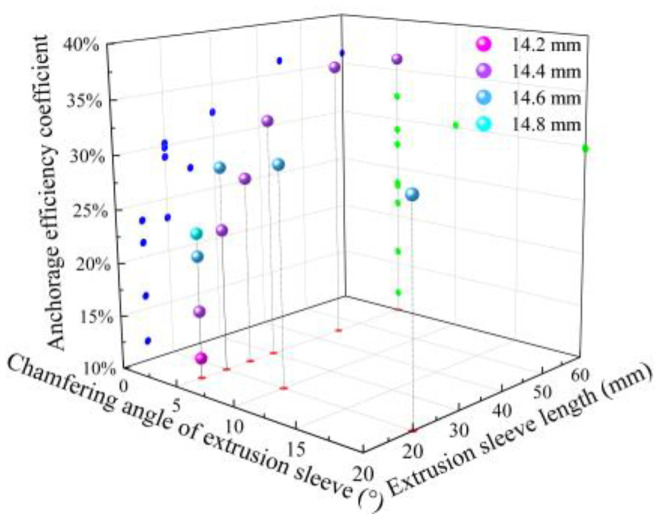
Static load test results of single extrusion sleeve.

**Figure 20:**
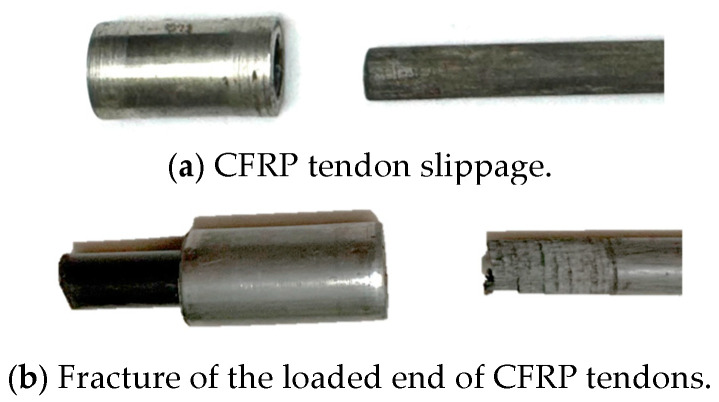
Failure mode of single extrusion sleeve.

The static tensile test results were used to calculate the anchorage efficiency coefficient using Equation (6), and the results are shown in Figure 19. Nine groups (D-1 to D-7, D-9 to D-10) exhibited CFRP tendon slippage from the extrusion sleeve, while three groups (D-8, D-11 to D-12) showed CFRP tendon breakage at the load-bearing end of the sleeve. Figure 20 presents the two different failure modes.

For extrusion sleeves with an outer diameter of Φ14.6 and a length of 20 mm, the anchorage efficiency initially increases and then decreases with chamfer angles of 5°, 10°, and 20°, as shown in Figure 19. This trend seems to contradict earlier finite element analysis, which suggested that lower chamfer angles are more effective. Analysis indicates that at chamfer angles of less than 10°, both the radial stress on the bar at the loaded end of the extrusion sleeve and the anchorage force provided by the sleeve are insufficient, failing to reach the bar’s breaking limit, thus resulting in slippage failure. As the chamfer angle increases, the overall radial stress on the bar, as shown in Figure 13, also increases slightly, thereby further enhancing the anchorage efficiency coefficient. However, when the chamfer angle increases to 20°, the radial stress on the bar at the loaded end significantly increases, causing the bar to reach failure conditions prematurely. Therefore, to optimize the anchorage efficiency of the extrusion sleeve, the chamfer angle should be minimized as much as possible.

For a 15 mm long extrusion sleeve with a 5° chamfer, the failure mode was tendon slippage across outer diameters of 14.2 mm, 14.4 mm, 14.6 mm, and 14.8 mm. As shown in Figure 19, anchorage efficiency increased with larger outer diameters, consistent with the finite element analysis in Section 4.2.

**Figure 21:**
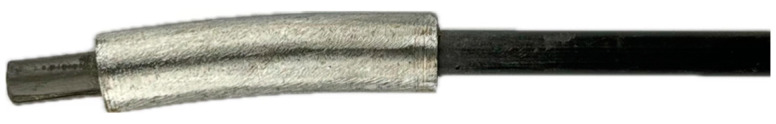
45 mm extrusion sleeve shape after extrusion.

For an extrusion sleeve with a 5° chamfer and a 14.4 mm outer diameter, tendon slippage occurred at lengths of 15 mm, 20 mm, 25 mm, and 30 mm. As shown in Figure 20, anchorage efficiency increased with longer sleeves. At lengths of 45 mm and 60 mm, the failure mode shifted to tendon breakage at the load-bearing end, and the anchorage efficiency coefficients were similar, as shown in Figure 19. Therefore, when the failure mode is tendon breakage at the load-bearing end, increasing the extrusion sleeve length has little effect on enhancing load capacity and may even reduce it. This aligns with the finite element analysis in Section 4.3, indicating that merely changing the sleeve length does not alleviate stress concentration at the load-bearing end and can cause tendon bending and damage during processing. The 45 mm extrusion sleeve after compression is shown in Figure 21.

### 6.2. Static Load Tensile Test of CFRP Tendons Anchored by Series Extrusion Sleeves

Given the low anchorage efficiency coefficient of single extrusion sleeves mentioned earlier, a new approach was proposed to use multiple extrusion sleeves in series to anchor a single CFRP tendon. Considering the failure modes and anchorage efficiency coefficients of single extrusion sleeves under different parameters, six specimens with various sleeve parameters were designed for static tensile tests. The parameters are listed in Table 3, with three identical specimens made for each parameter set.

**Figure 22:**
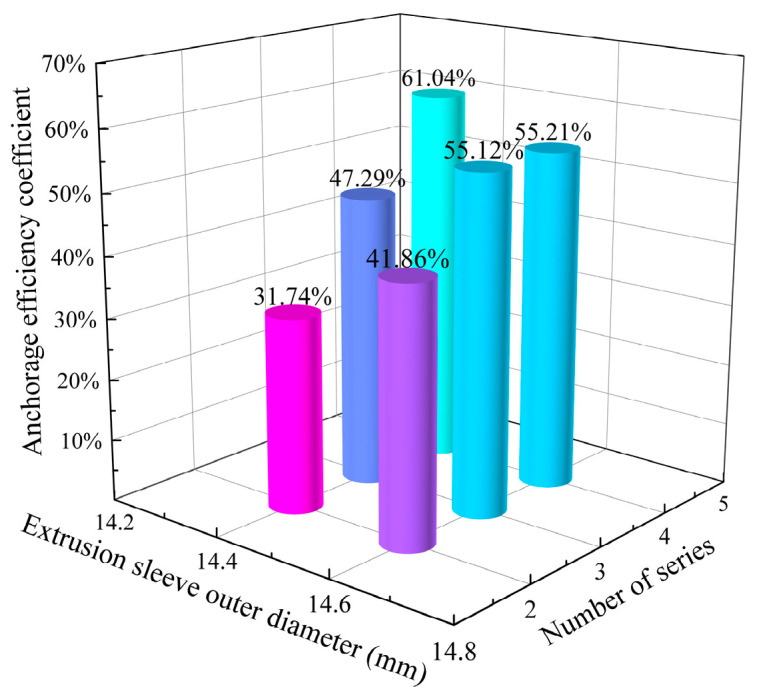
Static load test results of tandem extrusion sleeve.

**Figure 23:**
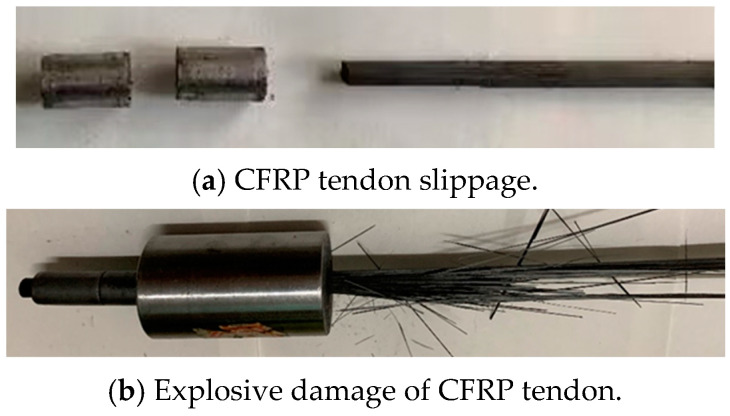
Failure mode of series extrusion sleeve.

The static tensile test results were used to calculate the anchorage efficiency coefficient using Equation (6), with the results shown in Figure 22. Specimens C-1 to C-3 exhibited CFRP tendon slippage from the sleeves, while specimens C-4 to C-6 showed explosive tendon failure. Figure 23 presents the two different failure modes.

Bar slippage failure occurs when the bar remains secure under the maximum axial and radial anchorage stresses exerted by the extrusion anchor at the loaded end. Conversely, explosive failure occurs when the number of extrusion sleeves in series increases, thereby enhancing the axial anchorage force. Consequently, the bar at the loaded end of the extrusion anchor is subjected to both axial and radial stresses, causing the external carbon fibers to reach their ultimate strength and fracture prematurely. At this point, the load surpasses the internal ultimate bearing capacity of the bar, resulting in explosive failure.

Analysis of the anchorage efficiency coefficient for sleeves with an outer diameter of Φ14.6, as shown in Figure 22, revealed that using two sleeves in series increased the coefficient by 15.48% compared to a single sleeve. However, increasing the number of sleeves from two to three only improved the coefficient by 13.26%, and from three to four, it increased by just 0.09%. This minimal increase is because the CFRP tendon reached its ultimate strength under radial stress at the load-bearing end, making further increases in the number of sleeves ineffective for significantly improving the anchorage efficiency.

When using sleeves with an outer diameter of Φ14.4, the anchorage efficiency coefficients were lower for two and three sleeves in series compared to Φ14.6 sleeves, due to reduced extrusion allowance. However, with four sleeves in series, the Φ14.4 sleeves showed better efficiency than the Φ14.6 sleeves, indicating that merely increasing extrusion allowance is not sufficient to enhance the anchorage effect on CFRP tendons.

When three or four extrusion sleeves, each with a length of 15 mm, an outer diameter of φ14.4 mm, and a chamfer angle of 5°, are connected in series, the anchorage length is consistent with that of a single sleeve with a length of 45 mm or 60 mm, an outer diameter of φ14.4 mm, and a chamfer angle of 5°. However, the anchorage efficiency coefficient is increased by 30.5% and 69.3%, respectively, demonstrating the rationality of the series anchorage structure proposed in this paper.

## 7. Conclusions

This paper proposes a series-connected extrusion sleeve anchoring structure for CFRP tendons. Finite element software was used to analyze the impact of various extrusion sleeve parameters on the radial stress distribution of the CFRP tendons in the anchorage zone, followed by static anchorage tests to validate the findings. The main conclusions are as follows:

(1) When the chamfer angle of the loaded end of the extrusion sleeve is smaller than the 10° taper angle of the extrusion die, the peak radial stress at the loaded end of the bar decreases rapidly with the reduction in the chamfer angle. Specifically, a chamfer angle of 5° results in a 21.4% reduction in peak radial stress compared to a chamfer angle of 20°. Therefore, a chamfer angle of 5° is recommended for the extrusion sleeve to optimize performance.

(2) In comparison to an outer diameter of Φ14.2, the average radial stress in the anchorage section increases by 29.6%, 55.5%, and 65.8% for extrusion sleeves with outer diameters of Φ14.4, Φ14.6, and Φ14.8, respectively. Similarly, the peak radial stress at the loaded end increases by 9.0%, 12.9%, and 18.7%. Increasing the outer diameter of the extrusion sleeve accelerates the bar’s premature attainment of its ultimate strength. Thus, considering both the peak radial stress at the loaded end and the corresponding anchorage efficiency coefficient, an outer diameter of Φ14.4 is deemed optimal for the extrusion sleeve.

(3) For extrusion sleeves with a chamfer angle of 5° and an outer diameter of 14.4 mm, the bar exhibits slippage failure at lengths of 15 mm, 20 mm, 25 mm, and 30 mm. As the length of the extrusion sleeve increases, the anchorage efficiency coefficient improves by 42.6%, 68.7%, and 98.7%, respectively, compared to the 15 mm sleeve. However, at lengths of 45 mm and 60 mm, the bar fails at the loaded end, and further increasing the sleeve length does little to improve the anchorage efficiency coefficient. Additionally, excessive sleeve length can damage the bar during processing, thereby reducing the anchorage efficiency coefficient. In contrast, using three or four 15 mm sleeves in series increases the anchorage efficiency coefficient by 30.5% and 69.3%, respectively, compared to a single sleeve of equivalent anchorage length. Therefore, a series of 15 mm long extrusion sleeves is recommended for anchorage.

(4) A series of four extrusion sleeves, each with a chamfer angle of 5°, an outer diameter of Φ14.4 mm, and a length of 15 mm, is effective for anchoring Φ7 CFRP bars. The results from static load tests indicate that the anchorage efficiency coefficient of the CFRP bars reaches 61.04%.

## Figures and Tables

**Table 1:** Material properties of each component.

Component	Material	Elastic Modulus (GPa)	Shear Modulus (GPa)	Poisson Ratio
CFRP tendon	CFRP	160	50.0	0.27
		10.3	50.0	0.27
		10.3	7.2	0.02
Extrusion die	Cr12MoV	210	-	0.30
Extrusion sleeve	45 steel	205	-	0.30

**Table 2:** Single extrusion sleeve anchorage CFRP bar extrusion sleeve parameters.

Test Piece	Chamfering Edge (°)	Outer Diameter (mm)	Length (mm)
D-1	5	14.2	15
D-2	5	14.4	15
D-3	5	14.6	15
D-4	5	14.8	15
D-5	5	14.4	20
D-6	5	14.6	20
D-7	10	14.6	20
D-8	20	14.6	20
D-9	5	14.4	25
D-10	5	14.4	30
D-11	5	14.4	45
D-12	5	14.4	60

**Table 3:** The parameters of the tandem extrusion sleeve for anchoring CFRP tendon extrusion sleeves.

Test Piece	Chamfering Edge (°)	Outer Diameter (mm)	Length (mm)	Number of Series
C-1	5	14.4	15	2
C-2	5	14.6	15	2
C-3	5	14.4	15	3
C-4	5	14.6	15	3
C-5	5	14.4	15	4
C-6	5	14.6	15	4

## Data Availability

The original contributions presented in the study are included in the article, further inquiries can be directed to the corresponding author.
